# COVID-19 Presenting as Major Thromboembolic Events: Virchow’s Triad Revisited and Clinical Considerations of Therapeutic Anticoagulation

**DOI:** 10.7759/cureus.10137

**Published:** 2020-08-30

**Authors:** Haoyi Zheng, Kathleen Stergiopoulos, Lin Wang, Lu Chen, Jane Cao

**Affiliations:** 1 The Heart Center, St. Francis Hospital, Roslyn, USA

**Keywords:** covid-19, thromboembolic event, virchow’s triad, hypercoagulable state, anticoagulation

## Abstract

This case series describes clinical course of three COVID-19 patients who presented with major thromboembolic events. Patient 1 is a 57-year-old male with asymptomatic COVID-19 who presented with a large left ventricular thrombus. His hospital course was complicated with a stroke. Patient 2 is a 71-year-old male with mild COVID-19 who presented with an acute stroke. Patient 3 is a 47-year-old male with severe COVID-19 who presented with a large pulmonary embolism. He died of a recurrent massive pulmonary embolism. This case series demonstrates that thromboembolic event can be the presenting feature of COVID-19 and can occur in the patients with asymptomatic or mild COVID-19. Diffuse endothelial injury and hypercoagulability play a pivotal role in recurrent thromboembolic events despite the anticoagulation. Therapeutic anticoagulation may be considered for severe COVID-19 patients and patients with important comorbidities or pre-existing endothelial dysfunction.

## Introduction

COVID-19 is the clinical manifestation of infection with severe acute respiratory syndrome coronavirus-2 (SARS-CoV-2). The respiratory distress including pneumonia and acute respiratory distress syndrome are the main clinical features. However, major thromboembolic complications including deep venous thrombosis, pulmonary embolism and stroke have been reported [1-3], which are attributed to the hypercoagulable state in COVID-19 patients. When present, thromboembolic events are associated with increased mortality [1, 4]. The mechanisms of hypercoagulable state are not fully understood. And the treatment with therapeutic anticoagulation remains controversial. So far, the majority of thromboembolic events have been reported in hospitalized patients with severe COVID-19. Failure of prophylactic anticoagulation in hospitalized COVID-19 patients has been reported [5]. Here we demonstrate three cases with asymptomatic, mild, and severe COVID-19 respectively who presented with major thromboembolic events in different organs. Following the case presentation we discussed the potential mechanisms and clinical considerations of therapeutic anticoagulation for patients with COVID-19. A waiver for individual consent was approved by the Institutional Review Board.

## Case presentation

Patient 1

**Figure 1:**
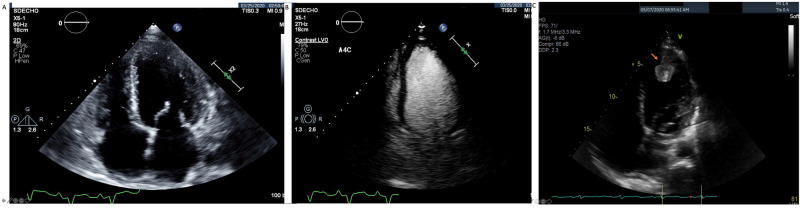
Left Ventricular Thrombus Formation in a Patient with Asymptomatic COVID-19 Initial transthoracic echocardiogram showed severely reduced left ventricular ejection fraction of 15-20% and global hypokinesis (A). There was no evidence of left ventricular thrombus in the contrast enhanced image (B). Subsequently, a large thrombus of 4.0 x 2.2 cm attached to the left ventricular apex is seen at the admission due to COVID-19 (C).

A 57-year-old male with a history of hypertension, diabetes, and recently diagnosed non-ischemic dilated cardiomyopathy, was admitted on May 6, 2020 due to increasing shortness of breath (SOB) and bilateral lower extremity edema. He had severely reduced left ventricular ejection fraction (LVEF) of 15-20% and the LV was free of thrombus from echocardiography done in March 2020 (Figure 1A, 1B).

**Figure 2:**
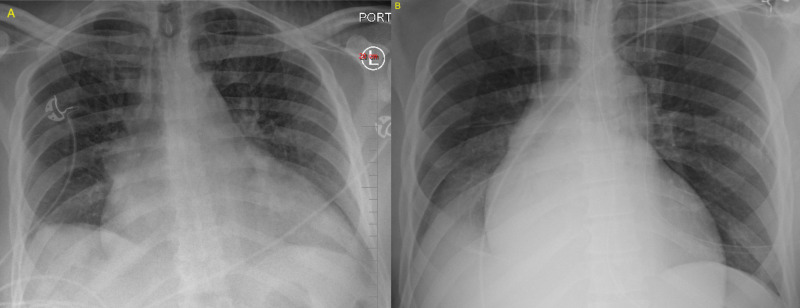
Chest Radiography on Admission and Prior to Discharge in a Patient with Asymptomatic COVID-19 Chest radiography on admission showed cardiomegaly, the cephalization of pulmonary vasculature from congestive heart failure, and no infiltrate (A). Chest radiography prior to discharge showed cardiomegaly, improved pulmonary vascular congestion, and no infiltrate (B).

On admission, the test for SARS-COV-2 was positive. He was afebrile and had no cough. His oxygen saturation was 97% with room air. His labs were not remarkable except elevated B-type natriuretic peptide (BNP) (11,463 pg/ml) and slightly elevated troponin (0.05 ng/ml). Echocardiography showed a large thrombus of 4.0 cm x 2.2 cm attached to the LV apex (Figure 1C). Lungs were free of infiltrate on chest radiography (Figure 2A). The patient was treated with intravenous heparin and oral Coumadin. On day 5, the patient had slurred speech, right-sided facial droop, and right-sided weakness and computerized tomography (CT) angiogram showed decreased filling of left middle cerebral artery circulation. He underwent emergent angiogram and thrombectomy. On day 11, the patient was discharged without any residual deficit from stroke. During his clinical course, the patient was without typical COVID-19 pneumonia (Figure 2B).

Patient 2

**Figure 3:**
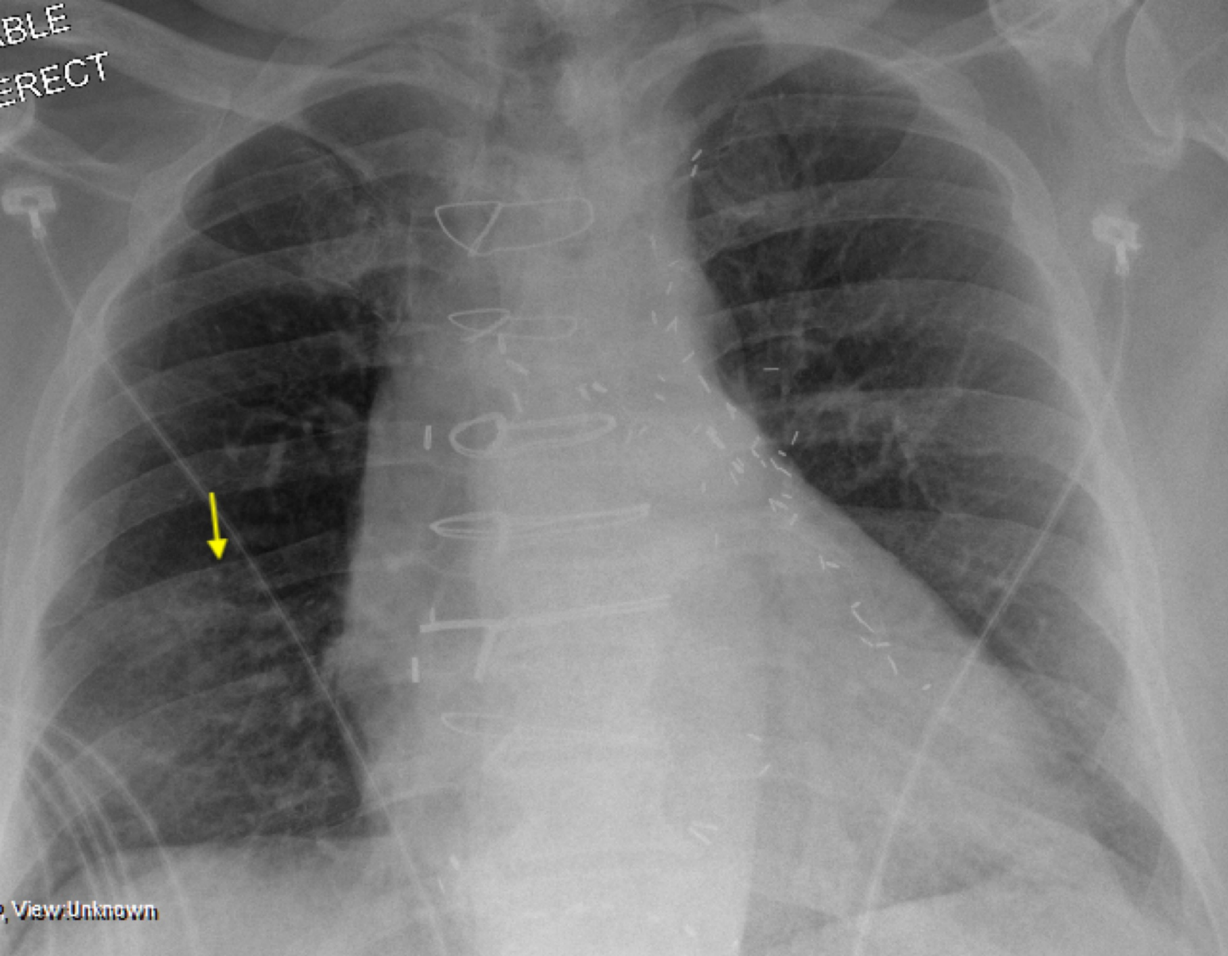
Chest Radiography of a Patient with Mild COVID-19 Chest radiography on admission showed patch infiltrate on right lower lobe.

**Figure 4:**
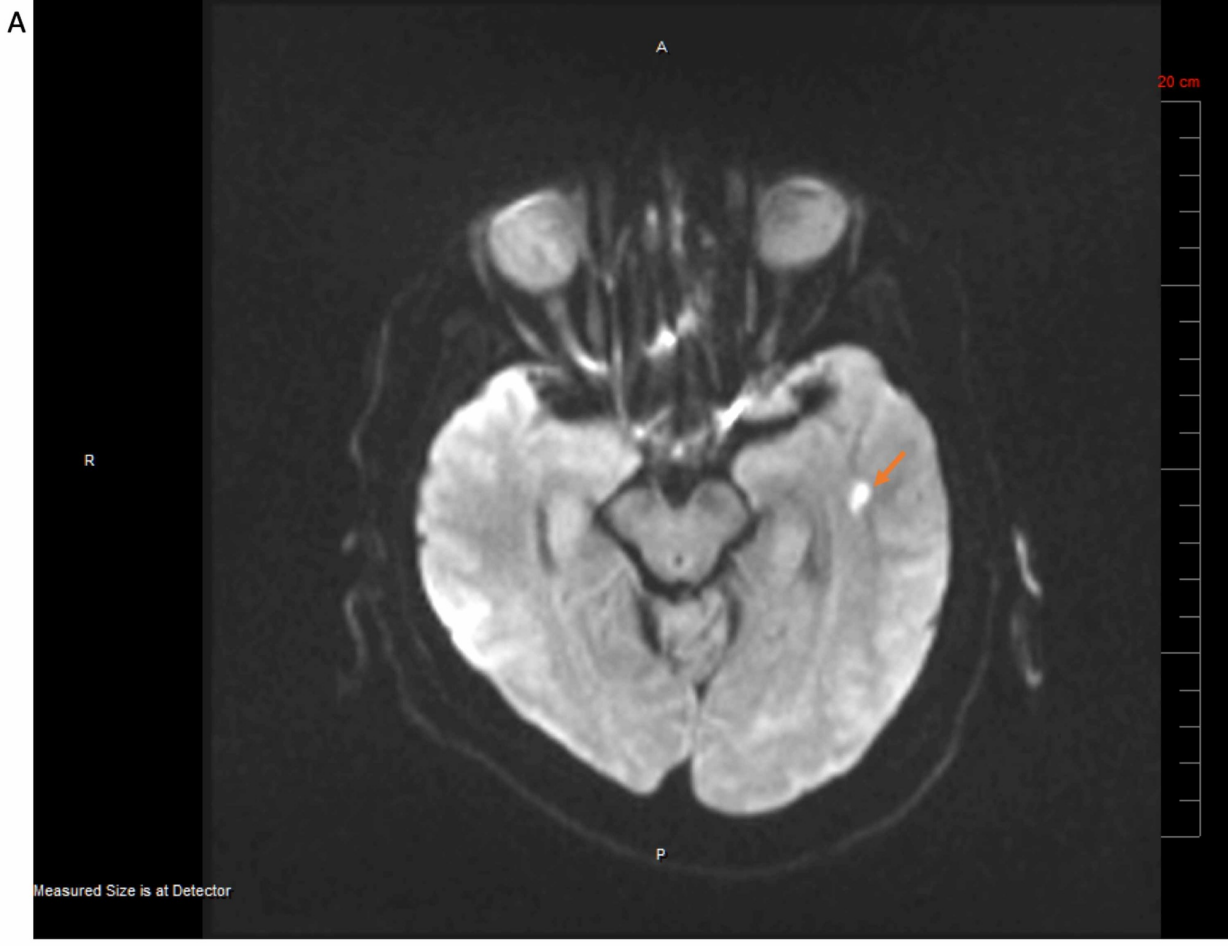
Acute Non-hemorrhagic Infarct in a Patient with Mild COVID-19 There is a small acute non-hemorrhagic infarct in the left anterior temporal lobe white matter on MRI.

**Figure 5:**
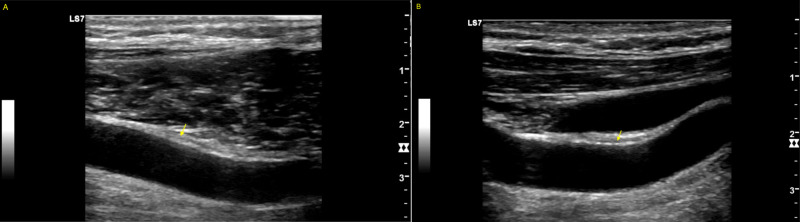
Carotid Artery Ultrasound for a Patient with Mild COVID-19 Carotid ultrasound showed no significant atheroma in the right carotid artery (A) and left carotid artery (B).

A 71-year-old male with a history of hypertension, diabetes, and coronary artery disease with recent coronary artery bypass graft (CABG) surgery presented with bilateral lower extremity weakness on April 17, 2020. He had chills, general myalgia, cough, and diarrhea for five days prior to the admission. On admission most of the symptoms resolved except for mild cough. His home medications included aspirin, rivaroxaban (2.5 mg twice daily), carvedilol, losartan, atorvastatin, and insulin. He was afebrile and his vital signs were normal. His oxygen saturation was 96% on room air. On physical examination his both lower extremities had 4/5 strength. Otherwise, there was no other neurological finding. The test for SARS-COV-2 was positive. There was patchy infiltrate in the right lower lobe on chest radiography (Figure 3). A diagnosis of an acute small non-hemorrhagic infarct in the white matter of the left anterior temporal lobe was established by magnetic resonance imaging (Figure 4). Carotid ultrasound showed less than 50% stenosis bilaterally (Figure 5A, 5B). The patient was treated with aspirin and rivaroxaban. On day 5, his bilateral lower extremity weakness improved and he was discharged to a rehabilitation facility.

Patient 3

**Figure 6:**
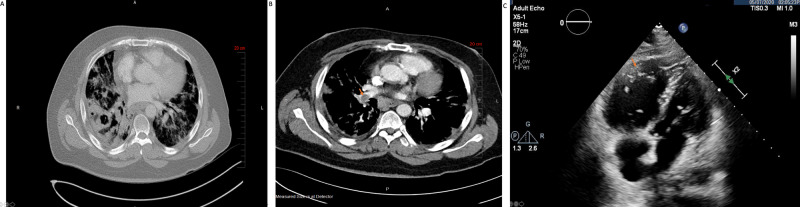
Pulmonary Thromboembolism in a Patient with Severe COVID-19 A large pulmonary embolism in the right pulmonary artery is seen in computerized tomography angiography which extends to the upper, lower, and middle lobar arteries and multiple segmental branches. In addition, there is a right lower lobe cavity lesion (A). There is diffuse bilateral ground glass-like interstitial airspace disease in the lungs (B). Transthoracic echocardiogram showing severely dilated right ventricle and severely decreased right ventricular systolic dysfunction (C).

A 47-year-old male with history of hypertension, diabetes, and peripheral vascular disease was admitted on May 5, 2020 for worsening shortness of breath (SOB) and chest pain. The patient presented to an outside hospital due to cough, fever, and SOB on April 3, 2020. The test for SARS-COV-2 was positive at that time. He was discharged and treated at home with hydroxychloroquine. Over the course of five weeks, he has had cough, SOB, and intermittent fever. On admission, the testing for SARS-COV-2 was again positive. A CT pulmonary angiography revealed a large thrombus in the right pulmonary artery and bilateral diffuse ground glass-like interstitial airspace disease in the lungs (Figure 6A, 6B). On admission, he was afebrile with a respiratory rate of 32 per minute, and oxygen saturation was 100% with a non-rebreather. Laboratory tests revealed elevated creatinine (2.9 mg/dL), partial-thromboplastin time (PTT) (61 seconds on heparin), ferritin (506 ng/ml), D-dimer (2980 µg/L), and fibrinogen (787 mg/dL). Lower extremity Doppler revealed right-sided deep venous thrombosis. Echocardiography showed a normal LV function but mildly reduced RV systolic function.

The patient was treated with intravenous heparin and received catheter-assisted thrombolytic with recombinant tissue plasminogen activator (r-tPA) on May 6, 2020. His symptoms were improved and oxygen requirement was reduced. On the following day, his hypoxia worsened and he became hypotensive and required intravenous vasopressors and mechanical ventilation. Echocardiography showed severely dilated RV and severely reduced RV systolic function (Figure 6C). An emergent thrombolytic therapy was given based on an empirical diagnosis of recurrent massive pulmonary embolism. However, the patient continued to deteriorate and soon developed cardiac arrest and died despite the resuscitation efforts.

## Discussion

In this case series we report thromboembolic complications in COVID-19 patients in the forms of LV thrombus, pulmonary embolism and ischemic stroke. The clinical presentations of COVID-19 varied significantly. Of the three cases presented, only case 3 had typical pneumonia related to COVID-19. Case 2 had mild respiratory symptoms and case 1 was asymptomatic from COVID-19. So our series demonstrate that major thromboembolic events can be the presenting feature of COVID-19 and can occur in asymptomatic or mild COVID-19. Recurrent thromboembolic event can occur even with therapeutic anticoagulation.

The direct causality between thromboembolic events and COVID-19 cannot be fully confirmed in this series due to the underlying comorbidities. However, these cases represent common scenarios of thromboembolic complications in patients with COVID-19. Reduced wall motion, local myocardial injury, and hypercoagulable state/stasis of blood flow are the Virchow’s triad in the ventricle for thrombus formation. The SARS-COV-2 infection with a resultant hypercoagulable state likely triggered a thrombus formation event in a dilated ventricle with severely reduced function. Similarly, blood flow alterations, endothelial injury, and hypercoagulability are the Virchow’s triad for the vascular thrombosis [6]. The hypercoagulable state with venous and arterial thrombotic events are common in hospitalized patients with COVID-19 [1,7].

The mechanism of hypercoagulability in patients with COVID-19 is not fully understood. COVID-19 patients show elevated D-dimmer, fibrinogen, and von Willebrand factor (VWF) [8]. Presence of lupus anticoagulant and factor XII deficiency have been reported [9]. Antiphospholipid antibodies were described in severe COVID-19 patients [10]. The potential roles of hypoxia and activated macrophage have been proposed [11, 12]. A direct injury of the endothelial cell, diffuse endothelial inflammation, and cardiac endotheliitis have been reported from autopsy [13, 14]. All these may contribute to the hypercoagulable state. As noted in this series, patient 1 further developed a stroke despite the treatment with the therapeutic heparin. Patient 2 was taking rivaroxaban and aspirin at home for coronary artery disease with recent CABG surgery prior to the stroke. Patient 3 had a recurrent massive pulmonary embolism despite the full anticoagulation with heparin and fibrinolytic therapy. Profound hypercoagulability and diffuse endothelial injury from SARS-COV-2 infection, two key elements of Virchow’s triad, may lead to recurrent thromboembolic events despite the intensive anticoagulation. These are consistent with reports that a high rate of thromboembolic events occurred in severe ICU COVID-19 patients despite therapeutic anticoagulation [7] and an increased rate of deep venous thrombosis in non-ICU patients hospitalized for COVID-19 despite thromboprophylaxis [5].

The current guideline recommends the use of prophylactic anticoagulation with low-molecular-weight heparin (LMWH) in all patients admitted with COVID-19 in the absence of contraindications [15]. With mounting evidence of thromboembolic risk associated with COVID-19 prophylactic anticoagulation seems insufficient and treating COVID-19 patients with therapeutic anticoagulation seems to be a reasonable choice. The initial anecdotal therapy showed improved outcome with anticoagulation [16]. A large retrospective study reported improved survival in hospitalized COVID-19 patients treated with anticoagulation [17].

Clinical considerations of therapeutic anticoagulation

To date, there is still lack of a randomized trial to confirm efficacy of therapeutic anticoagulation in COVID-19. It is imperative to develop a consensus to guide the clinical practice and improve patient outcomes. Based on the current observational data and our case series, we suggest that therapeutic anticoagulation may be considered for COVID-19 patients in the following situations:

1) Severe ICU patients requiring mechanical ventilation.

2) Non-ICU hospitalized patients with oxygen requirement and with pre-existing endothelial injury/dysfunction such as diabetes, peripheral vascular disease, active smoking, and severe obesity.

3) Mild COVID-19 patients with underlying ischemic and non-ischemic cardiomyopathy and severely decreased LV systolic function, who are treated or quarantined at home.

4) All patients with COVID-19 with history of deep venous thrombosis and pulmonary embolism.

Unfractionated heparin, therapeutic-dose LMWH, warfarin, and novel oral anticoagulants may be considered based on the clinical condition.

There are challenges despite the potential benefit of anticoagulation for COVID-19. A prolonged activated partial-thromboplastin time (aPTT) and factor XII deficiency have been reported as part of coagulopathy in patients with COVID-19 [8, 9]. These abnormalities may become barriers for patients to receive anticoagulation. However, limited but reassuring evidence suggests that prolonged aPTT was likely due to lupus anticoagulant and was not associated with a bleeding tendency upon receiving anticoagulation therapy [9]. Factor XII is not required for hemostasis thereby unlikely to contribute to bleeding risk. Therefore, clinicians should not withhold anticoagulation based on these abnormal parameters [9]. Furthermore, elevated fibrinogen level and other acute phase reactants in COVID-19 may cause heparin resistance [18]. All these should be kept in mind when considering the anticoagulation therapy in patients with COVID-19.

## Conclusions

Thromboembolic event can be a presenting feature of COVID-19 and can occur in asymptomatic or mild COVID-19. Recurrent thromboembolic event can occur despite therapeutic anticoagulation. Hypercoagulable state with profound endothelial injury following SARS-COV-2 infection plays a critical role in thrombosis. Due to the lack of efficacy and safety data of therapeutic anticoagulation from randomized trial in COVID-19, clinicians must balance the risk and benefit of the anticoagulation treatment and make the decision for each patient based on comorbidities and risk factors. Anticoagulation may be considered for ICU COVID-19 patients who require mechanical intubation, non-ICU patients with pre-existing endothelial injury/dysfunction, and patients with important comorbidities.

## Appendices

**Table 1:** Clinical Characteristics and Major Laboratory Findings at Admission of Three COVID-19 Patients Presented with Thromboembolic Events SARS-COV-2 = severe acute respiratory syndrome-coronavirus-2; SO2 = oxygen saturation; RT-PCR = reverse-transcriptase polymerase chain reaction; BNP = B-type natriuretic peptide; LV = left ventricle; NA = not available.

	Patient 1	Patient 2	Patient 3
Age, yrs	57	71	47
Sex	Male	Male	Male
Medical history	Hypertension, non-ischemic dilated cardiomyopathy	Hypertension, diabetes	Diabetes, peripheral vascular disease, chronic kidney disease
Medications at home	Spironolactone, sacubitril-valsartan, metoprolol succinate, metformin, furosemide	Aspirin, rivaroxaban, carvedilol, losartan, atorvastatin, insulin	Aspirin, insulin, oxycodone-acetaminophen, trazodone
SARS-COV-2 test	RT-PCR+	RT-PCR+	RT-PCR+
COVID-19 presentation	Asymptomatic No infiltrate	Chill, cough, and diarrhea, a small patchy infiltrate	Cough, fever, dyspnea, bilateral ground-glass infiltrates
COVID-19 treatment	No	No	Hydroxychloroquine
Presentation	Heart failure exacerbation and LV thrombus	Stroke	Pulmonary embolism
Heart rate (beats/min)	90	75	84
Respiratory rate (breaths/min)	26	18	32
Blood pressure (mm Hg)	135/90	125/80	110/64
SO_2_ (%, O2 requirement)	97 (room air)	96 (room air)	100 (10 L/min non-rebreather)
Temperature (ºC)	36.7	36	37.1
Total lymphocytes, 10^3^/mcl (0.5-3.1)	1.62	1.0	1.63
Platelet count, 10^3^/mcl (140-400)	255	151	386
Prothrombin time, s (9.4-11.2)	14	11.8	13.2
Partial thromboplastin time, s (23-32)	34	31	61 (on heparin)
Fibrinogen, mg/dl (209-495)	NA	NA	787
D-dimmer, mcg/l (0-400)	NA	NA	2980
High-sensitivity troponin I, ng/ml (0.01-0.03)	0.05	0.01	0.01
BNP, pg/ml (<100)	11463	NA	NA
C-reactive protein, mg/l (<3.0)	NA	NA	>180
Creatinine, mg/dl (0.70-1.33)	1.8	0.8	2.9
